# Physiotherapeutic Rehabilitative Approach in an Adult With Hemifacial Microsomia: A Case Report

**DOI:** 10.7759/cureus.28990

**Published:** 2022-09-09

**Authors:** Sakshi Kamani, Pooja Kasatwar

**Affiliations:** 1 Physiotherapy, Datta Meghe Institute of Medical Sciences, Wardha, IND; 2 Community Health Physiotherapy, Datta Meghe Institute of Medical Sciences, Wardha, IND

**Keywords:** interventions, hemifacial microsomia, pain, rehabilitation, physical therapy

## Abstract

Hemifacial microsomia is described as a congenital defect in which one-half of the face has an insufficient amount of skeletal and soft tissues. Underdeveloped temporomandibular joints, mandibular ramus, masticatory muscles, ears, and anomalies in face nerves and muscles are among the symptoms of this syndrome. We discuss the case of an 18-year-old young adult who has had facial asymmetry and malformation of the right ear from birth, as well as trouble chewing. Prior to surgery, the patient's mouth opening was restricted, and following surgery, the patient reported having trouble chewing and only a superficial mouth opening. A suitable and well-designed physical therapy programme was created for the patient in response to his clinical presentation, focusing on improving mouth opening and range of motion on the affected side. The purpose of this case study is to illustrate the major role therapeutic interventions can play in the treatment of hemifacial microsomia.

## Introduction

The underdevelopment of the bony jaws and soft tissues above them are the key features of the condition known as hemifacial microsomia; the ear is often, but not always, involved (microtia) [1]. Oculoauriculovertebral dysplasia is another name for HFM. The right side of the body seems to be impacted more frequently than the left, and men are more likely to be afflicted than women (3:2). If it occurs bilaterally, it is almost typically unilateral (70%) and asymmetrical [2].

According to Vento et al., each letter of the abbreviation O.M.E.N.S. represents one of the five key symptoms of HFM. The initials O, M, E, N, and S signify orbital distortion, mandibular hypoplasia, ear abnormalities, nerve involvement and soft tissue insufficiency, respectively. The OMENS system is a very flexible, all-inclusive, and essentially objective method of categorizing the spectrum of anomalies that make up hemifacial microsomia, making it one of the most thorough systems and as a result, most widely used to distinguish hemifacial microsomia. It includes issues with the facial nerve and extracranial structures in addition to skeletal and soft-tissue abnormalities [3-5].

Pruzansky's and Kaban's classifications are the most useful in clinical practice. Pruzansky created a scheme that gave a basic workable mandibular categorization based on three different types of mandibular abnormalities in a classic paper published in 1969. A grade I mandible, according to Pruzansky's method, is a tiny mandible with a normal temporomandibular joint (TMJ). It's basically a normal-shaped tiny mandible with a normal glenoid fossa and well-developed mastication muscles. Pruzansky's grade II mandible has a functional TMJ with a malformed condyle and a short, irregularly shaped ramus. The masticatory muscles are underdeveloped. Pruzansky's grade III mandible lacks the ramus, glenoid fossa, and TMJ - in other words, agenesis of the ramus [5]. The syndrome manifests itself in a variety of ways. There could be microtia or preauricular abnormalities in the ear that may range from severe to mild. In severe cases, it may result in facial palsy and hearing loss, also, ear deformity could be one of the major causes of HFM [6].

Significant facial deformities in HFM instances allow for easy differentiation from other craniofacial conditions. There are currently no universally accepted diagnostic criteria for this illness, despite the fact that multiple studies have demonstrated that either mandibular or ear abnormalities are required features. To rule out hemifacial microsomia, these minimum diagnostic criteria can be utilised: firstly, defects of the ipsilateral mandible and the middle ear; and secondly, a considerable family history or asymmetrical mandibular or ear (external/middle) deformities in conjunction with two or more anomalies that are indirectly related to them [7].

Hemifacial microsomia can be treated surgically and then further treated with orthodontic or orthotic treatment. In the case of hemifacial microsomia, introducing physical therapy measures might be advantageous to the patient [8]. To boost the patient's confidence and make him believe in getting promising results, interventions such as facial proprioceptive neuromuscular facilitation (PNF) to regain functions and actions of facial muscles, range of motion exercises to improve upper limb range of motion, strengthening exercises to improve strength, scar management, education to the patient about the condition, proper care about the condition, and home management could be given to him.

## Case presentation

An 18-year-old young adult male student from Pulgaon with an endomorphic build and right-handed dominance complained of trouble swallowing and mouth opening over the right side of his face at Acharya Vinoba Bhave Hospital Sawangi (M), Wardha, for a month before presentation. According to the patient, a previous operation was completed in 2006 in Acharya Vinoba Bhave Rural Hospital under general anaesthesia wherein costochondral grafting of the right temporal mandibular region was performed to treat his right-side hemifacial atrophy. In 2018, the patient underwent surgery to repair a facial deformity on the right side using a derma-fat graft, remove implants from the right mandibular region using the postage stamp technique on the buccal side, and reconstruct the defect using corticocancellous grafts from the right anterior iliac crest. Additionally, the patient underwent surgery to repair a right lateral cleft lip using primary closure while under general anaesthesia. The patient was scheduled for his third procedure on February 4, 2022, which included a bilateral Le Fort I osteotomy with a right maxilla anti-clockwise rotation by 7 mm, a right-side condylectomy with TMJ replacement, and mandibular body augmentation with specific implants under general anaesthesia. The patient received orthodontic care for one month; he was transferred to the ward for additional treatment the same day after his signs and symptoms were evaluated. To assist the patient regain the function and strength of his facial muscles, physiotherapy was also suggested.

Clinical examination

Extra-oral examination findings revealed evidence of deficient maturation on the right portion of the face, which appeared stubby and flattened with bottoming of the cheek, the body, and the ramus of the jaw on the right half compared to the left. This showed facial disproportion. The mandible significantly deviated to the right as the mouth opened and closed. When examined closely, the face exhibited asymmetry and has a smaller vertical and lateral mouth opening. In comparison with the left side, the TMJ movements (chewing, facial expressions) were relatively less on the correct side. The mandible also shows a pronounced deviation to the right. The chin was shifted to the right half of the face. The ear pinna was misshapen and minuscule. Lips were competent and no anomalies in the eyes, nose, mouth, ribs or skin were observed. The results of the intraoral examination show that the mouth opening was around 20 mm. Scar from prior surgery was visible in the retromolar area. Uvula, hard and soft palates were all reported NAD (no abnormality detected). Also, the buccal mucosa, tongue, and floor of the mouth were identified as NAD as well. 

Table 1* *lists the dates and events of the scheduled surgery, as well as the beginning of physiotherapeutic therapies.

**Table 1:** Timeline

Events	Dates
Patient admission	02/02/2022
Date of surgery	04/02/2022
Date of physiotherapy intervention	07/02/2022

Physiotherapeutic intervention

**Figure 1:**
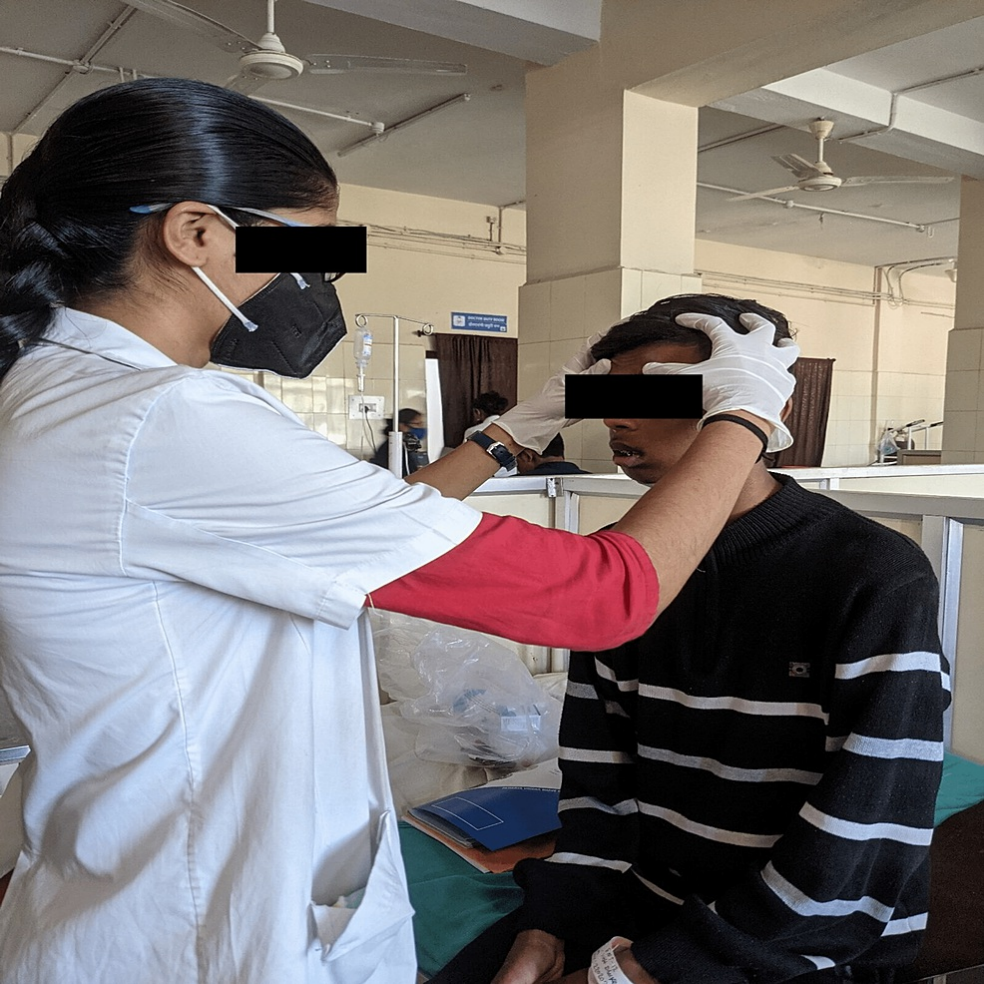
Facial PNF exercise demonstrated on the patient PNF: proprioceptive neuromuscular facilitation

A physiotherapy evaluation was performed on the first postoperative day (POD), and the patient complained of difficulty in mastication, limited upper extremity motions, inflamed areas of the neck, limited mouth opening, and other issues. A well-structured and goal-oriented physiotherapeutic plan was devised with the objective of correcting the patient's difficulties and achieving a functional and stable facial appearance using facial PNF techniques. Facial PNF helps in stimulating weakened facial muscles. It facilitates the action of facial muscles in functional facial movement and expression patterns. Also, it suppresses aberrant muscular activation interfering with the function of the face. At the conclusion of the three-week rehabilitation treatment, the patient was given a properly organized home programme, as shown in Table 2. It also lists the patient's goals, interventions, and regimen. As seen in Figure 1, the patient was given a facial PNF exercise.

**Table 2:** Physiotherapy rehabilitation

Goals	Physiotherapeutic Interventions	Regime
To educate the patient about the condition before starting rehabilitation.	Educating and counselling the patient, family members and caregivers.	The patient is educated and motivated throughout the session.
To improve mouth opening	(1) Active range of motion exercises for TMJ joints in all planes; (2) chin tucks; (3) resisted mouth opening.	Three times every day, the patient is recommended to execute a set of 15 repetitions.
To improve cervical joint mobility	Active range of motion exercises for the cervical joint.	A set of 10 repetitions is required of the patient twice daily.
To improve and strengthen shoulder joint musculature	(1) Active Full Range shoulder Movements; (2) pectoral stretches; (3) strengthening of shoulder joint using dumbbell/weight cuffs	A set of 10 repetitions is required of the patient twice daily. Two times every day, the patient must perform a set of 10 repetitions for strengthening.
To achieve an attractive and functional facial appearance	Facial PNF exercises that are guided consist of: (1) face centring exercises - suck your cheeks in between your teeth, take a long “ffff” sound and bring the brows together while blowing; (2) exercises for recovering eye closure - look down and close your eyes, keep looking down and close your eyes and squint them.	A set of 20 repetitions is required of the patient twice daily.
To avert and detach the adhered scar	Thumb kneading, massage and local squeezing are done to detach the adhered scar.	A 6-7 minute therapy session is given to the patient per day.
Home Programme	The patient is advised to perform and follow the above-mentioned protocol daily.	A follow-up update is necessary.

Prior to the patient being discharged, the patient underwent a complete examination using outcome measures after the three-week physiotherapeutic intervention programme. The successful outcomes following the rehabilitation protocol are shown in Table 3.

**Table 3:** Results ROM: range of motion; NPRS: Numerical Pain Rating Scale

Sr No.	Outcome measure		Pre-treatment		Post-treatment	
1	Mouth opening		Two complete fingers		Three complete fingers	
2	Pain (on NPRS)		8/10		1/10	
3	Chest expansion level	Axillary	1/3 cm		2/3 cm	
		Nipple	2/5 cm		3/5 cm	
		Xiphisternum	5/7cm		6/7 cm	
4	Cervical joint ROM	Rotation	Right	Left	Right	Left
			34	30	70	65
		Lateral flexion	30	30	50	45
5	Upper limb ROM	Shoulder flexion	120	170	160	175
		Extension	45	50	70	70
		Abduction	70	150	110	170
		Adduction	25	40	35	45
		Medial Rotation	40	85	70	90
		Lateral Rotation	30	50	50	60

**Figure 2:**
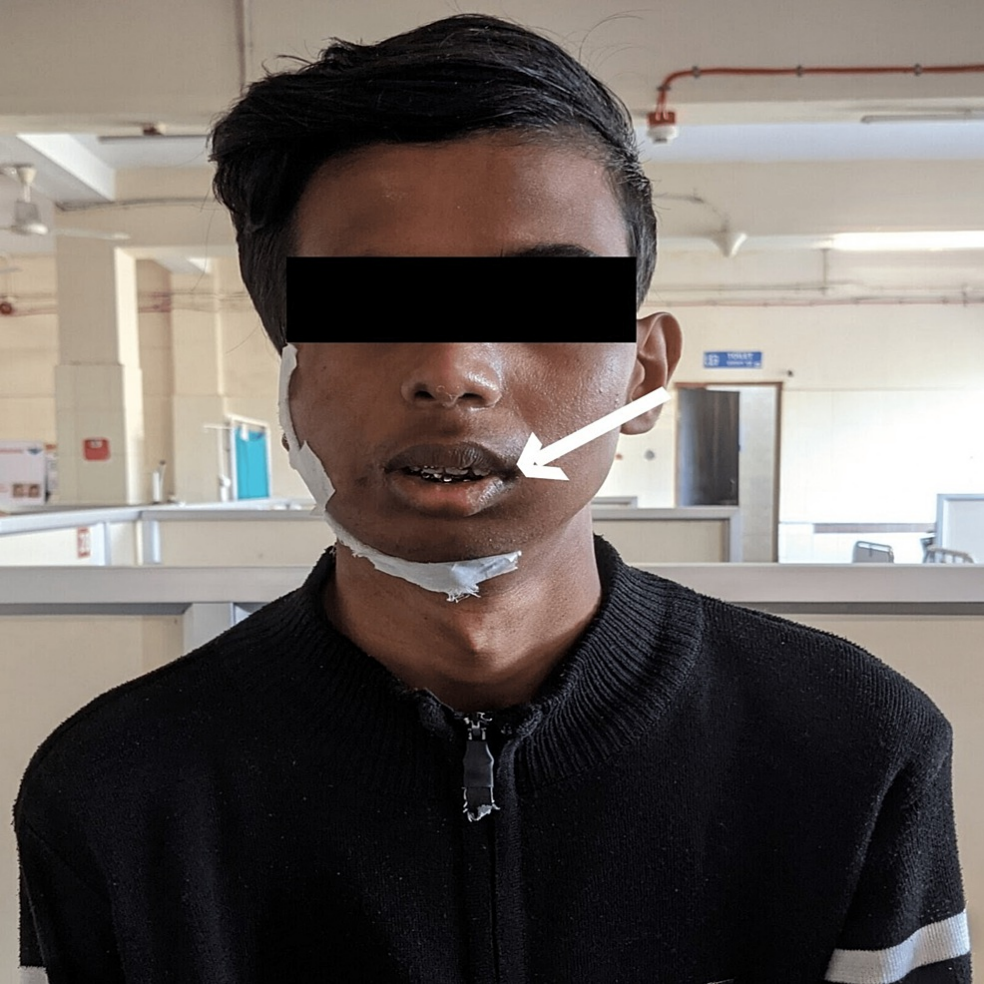
Mouth opening pre-treatment

**Figure 3:**
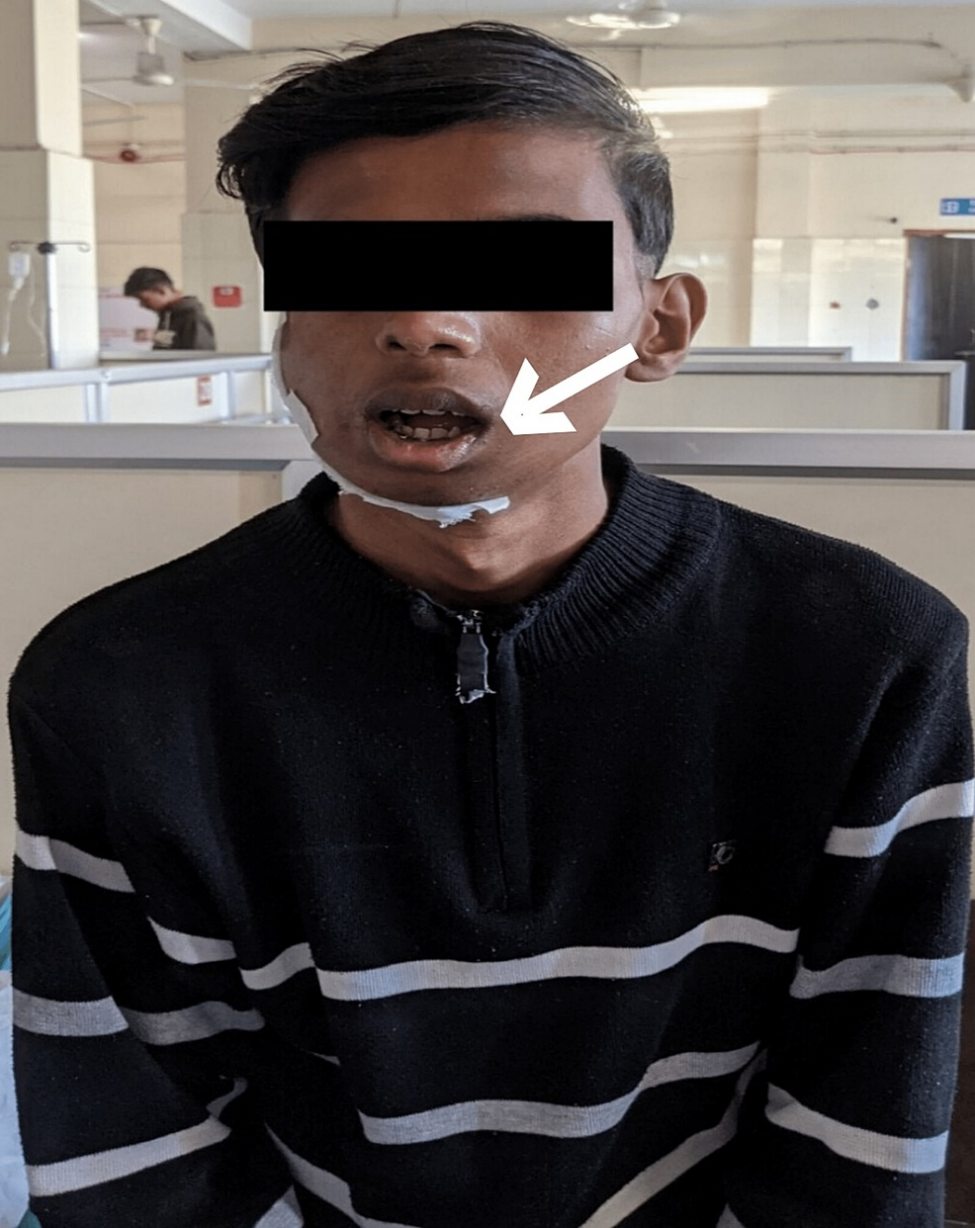
Mouth opening post-treatment

Following the outcome measurements, the patient's post-rehabilitation assessment was done. Figures 2-3 depict the progression of mouth opening before and after treatment. The progression delineates two complete fingers before treatment and three complete fingers after treatment.

## Discussion

Hemifacial microsomia was first used by Gorlin to refer to patients who had unilateral macrostomia, microtia, and failed mandibular ramus and condyle development [5]. Inadequate component development leads to asymmetrical congenital malformations of the head and face that are produced from the first and second branchial arches, which is the root cause of the HFM phenotypes. Even though "hemifacial" only refers to one-half of the face, the disorder is often bilateral and affects one side of the face more severely than the other in 31% of cases [9].

One of the most used systems for classifying hemifacial microsomia is the OMENS classification. The classifications developed by Pruzansky and Kaban, which classify mandibular anomalies into three categories, are the most frequently employed in medical care [3,5]. Anotia, a deformed pinna, and additional preauricular tags are among the ear anomalies. Because of the underdevelopment of the mandible, maxilla and malar bone, the face flattens. Microsomia can also be linked to a high-arch palate and a malocclusion of the teeth [10]. Only a small number of families have been found to have multiple sick individuals, both sexes, and transmission from male to male over several generations, all of which point to an autosomal dominant inheritance mechanism. The skin, subcutaneous tissues, mastication and expressive muscles, as well as the cranial nerves, may all be affected. Macrostomia is a condition that presents as a full-thickness lateral facial cleft. In severe cases, it may result in facial palsy and hearing loss; also, ear deformity could be one of the major causes of HFM.

## Conclusions

This case study highlights the importance of well-structured physical therapy therapies for pain alleviation, improvements in upper limb and cervical motions, and hospitalisation for postoperative patients with hemifacial microsomia. The patient showed significant improvement in mouth movements as well. Early use of the programme following surgery helps the patient recover from its side effects and quickly return to his social life.

## References

[1] Kapur R, Kapur R, Sheikh S, Jindal S, Kulkarni S (2008). Hemifacial microsomia: a case report. J Indian Soc Pedod Prev Dent.

[2] Cohen MM Jr, Rollnick BR, Kaye CI (1989). Oculoauriculovertebral spectrum: an updated critique. Cleft Palate J.

[3] Vento AR, LaBrie RA, Mulliken JB (1991). The O.M.E.N.S. classification of hemifacial microsomia. Cleft Palate Craniofac J.

[4] Gougoutas AJ, Singh DJ, Low DW, Bartlett SP (2007). Hemifacial microsomia: clinical features and pictographic representations of the OMENS classification system. Plast Reconstr Surg.

[5] Mielnik-Błaszczak M, Olszewska K (2011). Hemifacial microsomia - review of the literature. Dent Med Probl.

[6] Jackson IT (2004). Analysis and treatment of hemifacial microsomia. Eur J Plast Surg.

[7] Cousley RR, Calvert ML (1997). Current concepts in the understanding and management of hemifacial microsomia. Br J Plast Surg.

[8] Nouri M, Farzan A (2022). Nonsurgical treatment of hemifacial microsomia: a case report. Iran Red Crescent Med J.

[9] Shruthy R, Sharada P, Priya NK, Sreelatha HS, Jali PK, Suma MS (2017). Hemifacial microsomia - A case report and review. Niger J Exp Clin Biosci.

[10] Das A, Majumdar S, Chatterjee SS (1999). Atypical case of oculo-facio-auriculo-vertebral dysplasia (Goldenhar-Gorlin syndrome). Indian J Ophthalmol.

